# The role of dysregulated copper metabolism in diabetes and its complications: a review

**DOI:** 10.3389/fendo.2025.1681001

**Published:** 2025-11-05

**Authors:** Chen Wang, Junhong Wu, Yan Wang, Chengcheng Huang, Mengjuan Wei, Yufei Zhang, Renchu Shen, Jingwu Wang

**Affiliations:** ^1^ First Clinical Medical School, Shandong University of Traditional Chinese Medicine, Jinan, Shandong, China; ^2^ Affiliated Hospital of Shandong University of Traditional Chinese Medicine, Jinan, Shandong, China; ^3^ Beijing University of Chinese Medicine, Beijing, China

**Keywords:** Cu dysregulation1, cuproptosis2, DM3, mitochondrial proteolipid acylation4, lysosomal autophagy5

## Abstract

Copper (Cu) is an essential trace element for the human body. It significantly affects physiological and pathological processes by regulating various biological pathways, such as mitochondrial proteolipid acylation and glycolysis. Abnormal distribution, excess, or deficiency of Cu can trigger and accelerate the progression of diabetes mellitus (DM) and its complications through redox imbalance and activation of inflammatory pathways. In 2022, a novel form of programmed cell death termed cuproptosis was first identified by Peter Tsvetkov’s team. Increasing evidence indicates that patients with DM exhibit Cu dysregulation, suggesting that Cu dysregulation, exemplified by cuproptosis, might contribute to the pathogenesis of DM and its complications. Notably, regulating Cu metabolic homeostasis has demonstrated efficacy in delaying cancer progression. Similarly, preliminary studies on DM suggest that restoring Cu balance could ameliorate pathological cell death mediated by cuproptosis and oxidative stress. This approach represents a promising therapeutic strategy for DM and its associated complications. Therefore, this review summarizes recent advances regarding Cu dysregulation in DM patients, highlighting the significance of Cu homeostasis across multiple lesion sites associated with DM. Additionally, based on current evidence, this article discusses the regulatory role of Cu dysregulation in DM. Furthermore, we explore the potential molecular mechanisms underlying Cu dysregulation in DM, aiming to identify novel targets for therapeutic intervention.

## 1 Introduction

With changes in eating habits and lifestyle, the global incidence rate of diabetes mellitus (DM) has continued to rise in recent years. By 2021, the number of adults with DM (aged 20–79 years) reached approximately 537 million worldwide. This number is expected to increase by 46% by 2045 (1). Long-term glucose metabolism imbalance in DM patients can damage multiple tissues and organs, causing complications such as blindness, cognitive impairment, and peripheral neurovascular injury, seriously affecting patients’ quality of life (2). It may even lead to adverse clinical outcomes, such as cardiomyopathy and renal failure. The pathogenesis of DM is associated with mitochondrial dysfunction, chronic inflammation, and oxidative stress, all of which impair pancreatic β-cell function and induce insulin resistance (IR), causing absolute or relative insulin deficiency (3). Currently, DM treatment primarily includes hypoglycemic medications and insulin therapy. Symptomatic control is achieved by reducing sugar intake, delaying sugar absorption, promoting glucose clearance, and supplementing insulin (4). However, clinical efficacy for patients with insulin secretion deficiency (T1D) or insulin resistance (T2D) remains limited (5). Adverse effects, such as unstable glycemic levels, hypoglycemia, and gastrointestinal symptoms, frequently occur during treatment, significantly restricting patient eligibility. Therefore, further exploration of DM pathogenesis is critically important.

Copper (Cu), an essential cellular element, participates in mitochondrial respiration, antioxidant defense, and neurotransmitter synthesis (6). Cu cytotoxicity usually results from the strong pro-oxidant effects of unbound Cu^2+^ in mammalian tissues. Cu^+^ reacts with H_2_O_2_, generating highly reactive free radicals (HO^-^) that enhance lipid peroxidation, molecular damage, and cellular stress (7).

Cuproptosis, caused by excessive cytoplasmic Cu, is a distinct form of cell death frequently observed in tissues with high energy demands and abundant mitochondria. Unlike other known types of cell death, including necroptosis, pyroptosis, autophagy, and ferroptosis, cuproptosis results from Cu-mediated mitochondrial proteolipid acylation, causing DNA damage, lipid dysregulation, mitochondrial structural abnormalities, and dysfunction, ultimately leading to cell death (8) (**Table 1**). This process is often accompanied by substantial release of reactive oxygen species (ROS) and pro-inflammatory signaling molecules, excessively activating oxidative stress and inflammatory responses (51). The concept of cuproptosis originated from pan-cancer analyses. Its involvement in promoting tumor progression and its value in diagnosis, treatment, immune infiltration, and disease prognosis have been thoroughly demonstrated (52–54). Therefore, based on recent biological findings, this review emphasizes the significance of cuproptosis in DM and its complications and explores its potential diagnostic and therapeutic value. Increasing evidence suggests that Cu dysregulation, including Cu excess, Cu deficiency, and uneven Cu distribution, promotes cell dysfunction and death by disrupting antioxidant systems, cuproptosis, insulin secretion, and glucose metabolism, thereby significantly regulating the development of DM and its complications (55–58). For instance, the dysregulation of Cu transport ATPase A (ATP7A), ceruloplasmin (CP), superoxide dismutase 3 (SOD3), and other Cu proteins in pancreatic β-cells may lead to intracellular Cu accumulation, triggering increased ROS generation and causing pancreatic islet damage, ultimately resulting in DM (59). Cu deficiency in retinal tissues reduces the activities of antioxidant enzymes (SOD1), intensifying oxidative stress and causing retinal structural damage, which initiates diabetic retinopathy (60). Uneven Cu distribution, such as abnormal expression of Cu proteins (Cu transporter1 (CTR1) and SOD) in cardiomyocytes, reduces intracellular Cu^+^ and increases extracellular chelatable Cu^2+^, leading to excessive antioxidant consumption and initiating diabetic cardiomyopathy (DCM) (61). Therefore, this review focuses on the regulatory mechanisms of Cu metabolic homeostasis in DM and its complications, explores potential pathogenic mechanisms, and provides novel insights for targeted treatment strategies.

**Table 1:** Programmed cell necrosis mode.

Programmed cell death	Characteristics	Mechanism	Anomalous signaling molecule	References
Apoptosis	Cells form apoptotic vesicles	Physiological or external stimuli activate initial cysteine asparaginase (Caspase), loss of mitochondrial membrane potential, cell membrane phosphatidylserine (PS) ectopics, and DNA breaks	Caspase3、Caspase6、Caspase7、Bcl - 2 - associated X protein (BAX)、BCL - 2 homologous antagonist/killer (BAK)、 BCL - 2 - related ovarian killer (BOK)、Tumor Necrosis Factor (TNF)、Programmed Cell Death Protein 53 (P53)、ROS	(9–12)
Pyroptosis	Activation of inflammatory vesicle complexes	Intracellular and extracellular stimuli activate inflammatory vesicles, and Gasdermin family proteins damage the cell membrane to the point of rupture, followed by the release of cellular contents	Nucleotide - binding oligomerization domain - like receptor family pyrin domain - containing 3 (NLRP 3)、Interferon - inducible protein 16 (AIM 2)、Pyrin domain - containing protein (PYRIN)、Caspase 1、Caspase 3、Gasdermin family、ROS	(13–17)
Ferroptosis	Iron overload, lipid peroxidation	Intracellular Fe^2+^ hoarding triggers the Fenton reaction, causing lipid peroxidation, massive ROS generation, and the production of cytotoxic hydroxyl radicals, leading to mitochondrial morphology and functional abnormalities	Cystine - glutamate antiporter system xc -、GSH、Glutathione Peroxidase 4 (GPX4)、P53、P62、acyl - CoA synthetase long - chain family member 4 (ACSL 4)、Solute Carrier Family 7 Member 11 (SLC 7A11)、Ferritin Heavy Chain 1 (FTH 1)、Fibroblast Specific Protein 1 (FSP 1)、Nrf2、Heme Oxygenase – 1 (Ho-1)、ROS	(18–21)
Cuproptosis	Copper overload, mitochondrial proteolipid acylation and protein oligomerization	Mitochondria receive excess cytoplasmic copper and the TCA cycle lipoyl protein pyruvate dehydrogenase complex aggregates, inducing mitochondrial metabolic dysfunction and acute proteotoxic stress	DLAT、PDHA1、PDHB、CTR、SOD、MT、CcO、GSH、ATP7A、ATP7B、CP、TP53、ROS	(6–8, 22–47)
Disulfidptosis	Collapse of cytoskeleton proteins	Massive accumulation of intracellular disulfide-bonded molecules triggered by glucose starvation, increased galactose levels, and subsequent DPP depletion, F-actin cytoskeleton damage	SLC 7A11、Nicotinamide Adenine Dinucleotide Phosphate Hydrogen (NADPH)、Filamentous actin、Rho - associated coiled - coil - forming protein kinase (ROCK)、Peroxiredoxin - 1、Cysteine - 173	(21, 48–50)

## 2 Molecular mechanisms of copper metabolism

In the human body, Cu ions enter and exit cells via plasma membrane proteins, including CTR, metallothionein (MT), SOD, glutathione (GSH), ATP7A, ATP7B, and CP, exerting biological functions through organelles such as mitochondria and lysosomes (62). Cu homeostasis is maintained by cellular uptake, transport, sequestration, and excretion (63). Abnormal expression of Cu-associated proteins and transporters may lead to Cu excess, Cu deficiency, and uneven Cu distribution. Excessive Cu can induce mitochondrial proteolipid acylation, causing organelle dysfunction or even cell death, which mediates tissue damage (8). This section summarizes physiological Cu metabolism, analyzes the mechanisms underlying Cu dyshomeostasis, and discusses potential pathogenic pathways.

### 2.1 Physiological processes of copper metabolism

**Figure 1:**
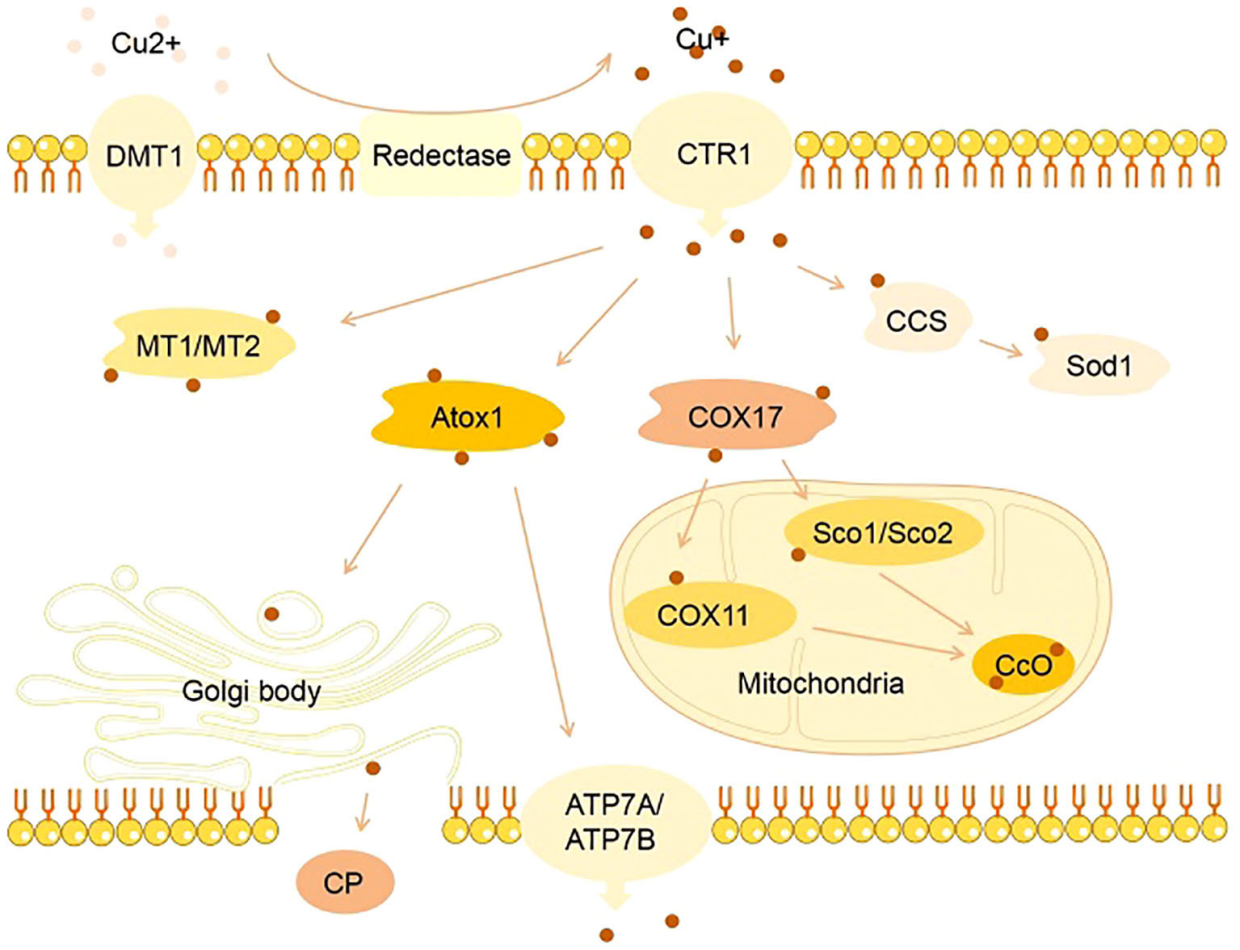
Physiological processes of Cu metabolism. Cu homeostasis depends on precise regulation by multiple proteins. Under physiological conditions, extracellular Cu^2+^ binds to divalent metal transporter 1 (DMT1) and enters intestinal cells. Simultaneously, Cu^+^ reduced by the six-transmembrane epithelial antigen of prostate (STEAP) family is transported into cells via CTR1-mediated endocytosis. Intracellular Cu^+^ binds to chaperone proteins such as antioxidant protein 1 (Atox1) and MT in the cytoplasm. The CTR1- copper chaperone for superoxide dismutase (CCS) -SOD1 complex delivers Cu^+^ to SOD1, whereas Cu chaperone for cytochrome c oxidase 17 (COX17) transports Cu^+^ to mitochondrial cytochrome c oxidase (CCO) through synthesis of CCO 1/2 (Sco1/2), participating in energy metabolism. Under specific conditions, transcription factors such as metal transcription factor 1 (MTF1) and nuclear factor erythroid 2-related factor 2 (Nrf2) mediate the Atox1/ATPase pathway, transferring Cu^+^ to the Golgi to form CP, which delivers Cu to target organelles or secretes it into the bloodstream. ATP7A and ATP7B regulate Cu excretion, controlling the systemic distribution of Cu ions. Cu binds cyclically to CP, albumin, and free amino acids, reaches the liver and other organs, and is reabsorbed via CTR1, regulating physiological processes.

Cu homeostasis depends on precise regulation by multiple proteins (**Figure 1**). Under physiological conditions, extracellular Cu^2+^ binds to divalent metal transporter 1 (DMT1) and enters intestinal cells. Simultaneously, Cu^+^ reduced by the six-transmembrane epithelial antigen of prostate (STEAP) family is transported into cells via CTR1-mediated endocytosis (64). Proteins involved in Cu influx are negatively regulated by intracellular Cu concentrations. When intracellular Cu increases, CTR1 undergoes endocytic degradation, reducing further Cu uptake, and vice versa (65). Studies showed that intestinal-specific knockout of CTR1 in mice caused severe systemic Cu deficiency and death within approximately 20 days after birth, while global knockout resulted in embryonic lethality (66), highlighting the crucial role of CTR1 in Cu uptake. Intracellular Cu^+^ binds to chaperone proteins such as antioxidant protein 1 (Atox1) and MT in the cytoplasm (67). The CTR1- copper chaperone for superoxide dismutase (CCS) -SOD1 complex delivers Cu^+^ to SOD1 (68), whereas Cu chaperone for cytochrome c oxidase 17 (COX17) transports Cu^+^ to mitochondrial cytochrome c oxidase (CCO) through synthesis of CCO 1/2 (Sco1/2), participating in energy metabolism (69). Under specific conditions, transcription factors such as metal transcription factor 1 (MTF1) and nuclear factor erythroid 2-related factor 2 (Nrf2) mediate the Atox1/ATPase pathway, transferring Cu^+^ to the Golgi to form CP, which delivers Cu to target organelles or secretes it into the bloodstream (70). ATP7A and ATP7B regulate Cu excretion, controlling the systemic distribution of Cu ions (71). Cu binds cyclically to CP, albumin, and free amino acids, reaches the liver and other organs, and is reabsorbed via CTR1, regulating physiological processes (72). Mutations in ATP7A/ATP7B genes cause cellular Cu overload and toxic effects in mouse models (73). Thus, Cu chaperones and transporters are key regulators of Cu homeostasis in various tissues. Abnormalities in these proteins disrupt Cu balance, triggering disease cascades (74).

### 2.2 Cu dysregulation and mitochondrial respiration

Cu exerts high cytotoxicity through two main mechanisms (75). First, it regulates electron transfer, exhibiting potent activity in redox reactions (22). Second, free Cu ions generate intracellular ROS (23). During Cu^+^/Cu^2+^ transitions, electrons are transferred via the Fenton reaction, producing ROS, such as ROS, O^2-^, NO^-^, OH^-^, and H_2_O_2_. This process leads to lipid peroxidation, proteolipid acylation, nucleic acid damage, and ultimately, cell death (24).

Normal mitochondrial respiration is critical for maintaining Cu homeostasis through its regulation of Cu chaperones and transporters (76). Tsvetkov et al. proposed that mitochondrial proteolipid acylation is the central mechanism underlying cuproptosis (8). Cells highly dependent on mitochondrial respiration are nearly 1000-fold more sensitive to Cu ion carriers compared to cells relying on glycolysis. This phenomenon may relate to high levels of lipoylated enzymes in the tricarboxylic acid (TCA) cycle, emphasizing mitochondrial respiration’s role in cuproptosis. Mitochondrial respiration promotes intracellular Cu accumulation, characterized by excessive mitochondrial Cu uptake. This uptake results in aggregation of lipoylated TCA cycle enzymes, particularly pyruvate dehydrogenase complexes (dihydrolipoamide S-acetyltransferase (DLAT), pyruvate dehydrogenase E1 subunit α1 (PDHA1), and pyruvate dehydrogenase β subunit (PDHB)), as direct Cu-binding targets, accompanied by loss of iron-sulfur cluster proteins (77). Disulfiram-Cu^2+^ complexes inhibit ubiquitinated protein degradation through ATP-synthase-dependent ubiquitination pathways, induce mitochondrial metabolic dysfunction, acute proteotoxic stress, and ultimately cause cuproptosis (25). When mitochondrial Cu is depleted, cellular metabolism shifts toward glycolysis, increasing ROS generation and reducing oxidative phosphorylation (26). ROS damage proteins, nucleic acids, and lipids, disrupt iron-sulfur cluster synthesis (27), and alter cellular energy metabolism. Zhang et al. found that glucose transporter inhibitor phloretin (Ph) promotes lipoprotein aggregation by blocking glucose uptake and glycolysis, enhancing cuproptosis sensitivity in mouse colon cancer cells, thus exerting anticancer effects (28). Therefore, cuproptosis is closely associated with inhibited glycolysis, suggesting metabolic intervention as a potential therapeutic approach for various diseases.

### 2.3 Cu excretion and lysosomal autophagy

Lysosomal autophagy, a protective mechanism, clears abnormal proteins and damaged organelles through lysosomes. It mitigates Cu-induced lipid accumulation, maintains cellular Cu homeostasis, and prevents Cu-induced apoptosis (29). The liver plays a pivotal role in Cu storage and metabolic regulation. Under physiological conditions, Cu enzymes in different cytoplasmic compartments are oxidized by CP (78). Upon increased Cu levels, hepatic ATP7B moves from the Golgi apparatus to lysosomes. Excess Cu binds to cytoplasmic MTs or is stored in lysosomes. Lysosomal ATP7B interacts with p62, triggering exocytosis and releasing Cu into bile (79). Under pathological conditions, loss of ATP7B function causes increased autophagic structures in the cytoplasm, positively correlated with cytoplasmic Cu levels. Excessive Cu promotes lysosomal-autophagosome fusion for degradation. Reduced activity of mechanistic target of rapamycin (mTOR) activates autophagy-related genes, preventing cuproptosis (29). Lysosomes regulate proteostasis and endoplasmic reticulum stress via autophagy (30). Their functions are regulated by transcription factors, including transcription factor EB (TFEB), transcription factor binding to immunoglobulin heavy constant mu enhancer 3 (TFE3), and activation of mTOR complex 1 (mTORC1) and mTORC2 (31, 80, 81). Thus, lysosomal autophagy is crucial in coordinating Cu homeostasis and preventing secondary injury. However, ATP7A deficiency in intestinal cells can impair lysosomal function, blocking the autophagic clearance of excess Cu ions and limiting therapeutic effectiveness (32). Therefore, ATP7A remains essential for maintaining intracellular Cu homeostasis and autophagic balance.

## 3 Role of Cu dysregulation in DM and its complications

**Figure 2:**
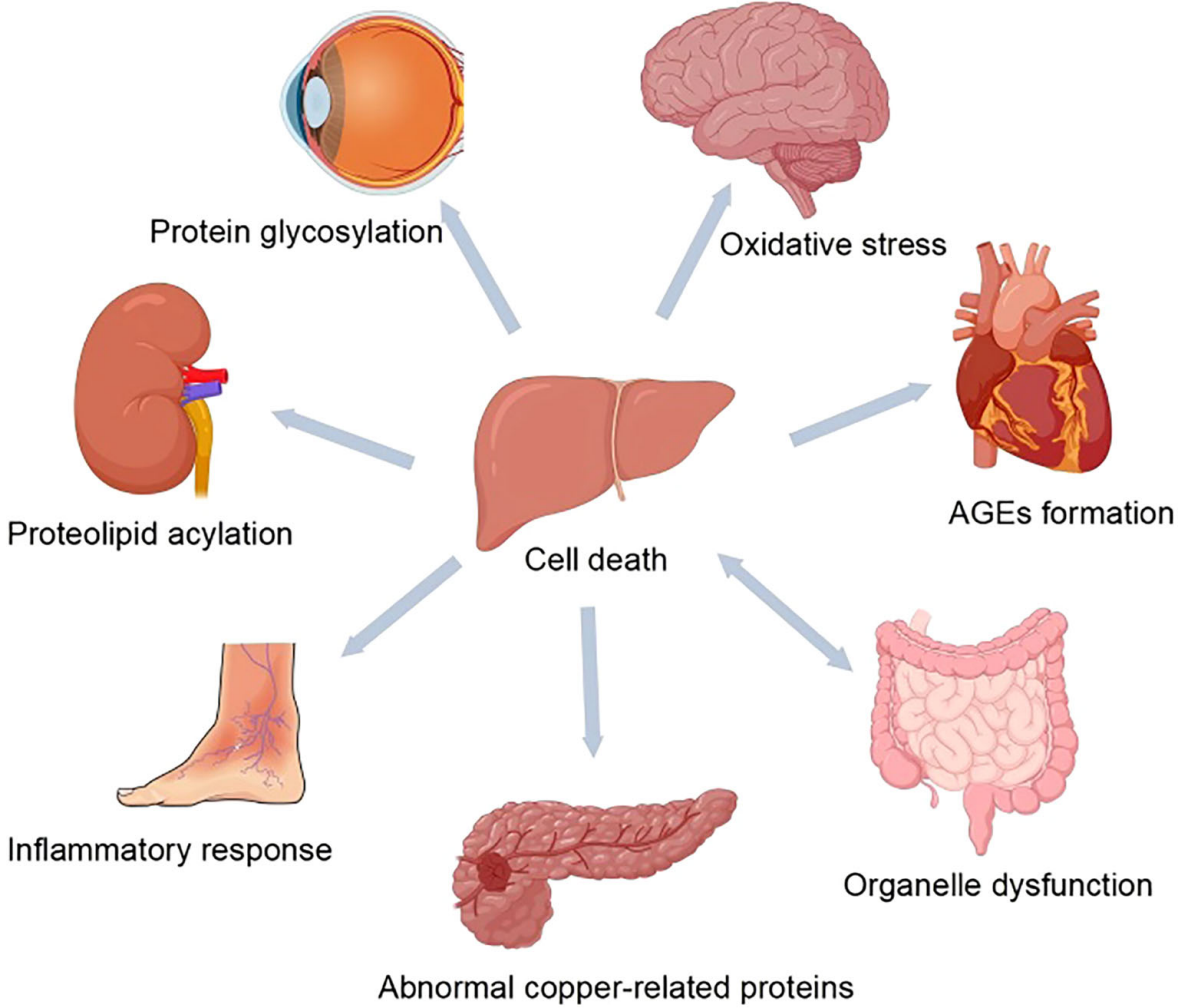
The role of dysregulated copper metabolism in diabetes and its complications. Cu dysregulation results from abnormalities in Cu chaperone and transport proteins. By mediating oxidative stress imbalance and upregulating inflammatory responses, it promotes the release of large amounts of ROS and inflammatory factors, damaging pancreas and target organs such as heart, kidney and retina. In addition, it disrupts oxidative phosphorylation–mediated cuproptosis and metabolic reprogramming through mitochondrial proteolipid acylation, thereby impairing insulin and energy metabolism.

Cu dysregulation results from abnormalities in Cu chaperone and transport proteins. By mediating oxidative stress imbalance and upregulating inflammatory responses, it promotes the release of large amounts of ROS and inflammatory factors, damaging pancreatic β-cells. In addition, it disrupts oxidative phosphorylation–mediated cuproptosis and metabolic reprogramming through mitochondrial proteolipid acylation, thereby impairing insulin and energy metabolism (58, 82, 83) and accelerating the progression of DM and its complications (57, 84). Conversely, abnormal blood glucose levels can exacerbate Cu dysregulation by triggering glycation reactions (85). Therefore, this section focuses on the pathological damage, molecular mechanisms, and prognostic impact of Cu dysregulation in DM and its complications (**Table 2**) (**Figure 2**).

**Table 2:** Molecular mechanisms of copper in various types of diabetes and complications.

Diabetes type	Clinical implications	Cu status	Mechanism	Reference
T2D	Abdominal obesity, insulin deficiency, IR	Cu overload in serum	Aberrant expression of copper proteins leads to copper accumulation, stimulates oxidative stress, cuproptosis, and disrupts insulin and glucose metabolism	(86–98)
T1D	Absolute insulin deficiency	Cu overload in serum		(96, 99–105)
GDM	Excessive weight gain	Cu overload in serum, Cu deficiency with low BMI		(106–111)
DCM	Myocardial fibrosis, dilated cardiomyopathy	Excess serum Cu, Cu deficiency in cardiomyocytes	Inadequate myocardial copper uptake, extracellular cumulative copper toxicity, decreased connective tissue toughness and elasticity, and compensatory myocardial contraction	(58, 112–121)
DKD	Hypertension, kidney failure	Excess Cu deposits in kidney tissue	Copper excess induces oxidative stress and altered glomerular basement membrane charge selectivity	(122–131)
DR	Blurred vision, blindness	Cu excess and deficiency	Excess serum copper and insufficient intracellular copper stimulate oxidative stress and damage to the retinal pigmented epidermis, plexiform layer and optic nerve fibers	(85, 132–137)
DPVD	Vascular sclerosis, occlusion	Cu overload in serum	Excess serum copper promotes fibrosis and highly glycosylated elastin and collagen within the arterial wall	(92, 114, 138–143)

### 3.1 Cu dysregulation and DM

#### 3.1.1 T2D

T2D is a chronic metabolic disorder characterized by insulin resistance and pancreatic β-cell dysfunction. A positive correlation between circulating Cu content and blood glucose has been reported. Meta-analyses and generalized linear model (GLM) studies involving tens of thousands of participants have consistently shownthat Cu levels in peripheral blood and urine are positively correlated with fasting blood glucose (FBG) and glycated hemoglobin (HbA1c), and negatively correlated with C-peptide levels and β-cell function (86, 87, 144). Lower Cu concentrations exert a protective effect on pancreatic β-cells in T2D patients. These findings suggest that reducing excess Cu may improve β-cell function and glucose metabolism in DM, representing a potential therapeutic strategy.

However, a cross-sectional study in healthy adults found that high dietary Cu intake was associated with reduced insulin and homeostatic model assessment (HOMA) of IR index (88). This only indicates that moderate Cu intake may lower insulin levels; it does not establish a pathological negative association. Large-scale, long-term studies are needed to confirm whether increased Cu intake reduces T2D incidence.

Several mechanisms have been proposed for Cu dysregulation in T2D. Cu protein imbalance induces excessive oxidative stress, damaging pancreatic β-cell function and mediating cuproptosis, thereby disrupting blood glucose homeostasis. Inactivation of CP and decreased ATP7A expression in pancreatic β-cells impair SOD3 metallation within the trans-Golgi network, inhibiting Cu efflux and causing intracellular Cu accumulation (89). Copper-containing complexes have been demonstrated to promote the deposition of human insulin (HA) through the Haber-Weiss and Fenton reactions, generating H_2_O_2_ (90). These complexes also participate in ROS production through hydrogen extraction catalysis, increasing oxidative stress, causing damage to pancreatic β-cells, and leading to insulin deficiency or resistance (IR), ultimately resulting in diabetes (91–93).

Cuproptosis has been proposed as a potential pathogenic mechanism of DM (145). Excess Cu binds to acylated proteolipids, causing oligomerization and functional loss. This leads to aggregation and inactivation of proteins such as pyruvate dehydrogenase complex (PDC), PDHA1, and PDHB. During this process, the ATP/ADP ratio rises, closing ATP-dependent potassium channels and opening calcium channels on the plasma membrane, triggering extracellular insulin secretion. This process is regulated by PDC, and metabolic coupling amplifies insulin secretion (146). In DM animal models, PDC activity is significantly reduced (147), leading to glucose metabolic disorders, β-cell dysfunction, and impaired insulin release. PDHA1 knockout mice (bKO) display glucose intolerance and markedly reduced pancreatic secretion but no decrease in insulin sensitivity (148), indicating that hyperglycemia in bKO mice results from β-cell dysfunction and insulin secretion imbalance rather than peripheral tissue insulin resistance. This confirms the critical role of PDHA1 in pancreatic islet development, as its absence markedly alters islet morphology and function (148), highlighting the direct damaging effects of excess Cu on β-cells.

*In vivo* and *in vitro* experiments also show that excess Cu interferes with glycolytic enzyme activity, hindering glycolysis and leading to metabolic disorders and DM (149). Cu participates in glucose oxidative phosphorylation (150), regulating cellular glucose uptake and plasma glucose levels. Cu increases glucose transporter 1 (GLUT1) expression via the hypoxia-inducible factor 1α (HIF-1α)-dependent pathway, similar to the function of insulin-degrading enzyme (IDE) in mouse models (151). PDHB, a novel DM risk gene (152), promotes transcription of glycolysis-related genes by binding to solute carrier family 2 member 1 (SLC2A1) and other promoters, mediating metabolic reprogramming (153). Increased phosphorylation of PDHA1 E1 subunit inhibits fatty acid metabolism and proteolipid acylation (154), suppresses the TCA cycle, and diverts acetyl-CoA and α-ketoglutarate to amino acid synthesis (155), causing incomplete glucose oxidation and limiting glucose metabolism. This provides a new perspective on metabolic reprogramming in T2D. However, in the cited study, glucose metabolism was represented by metabolic flux, which is difficult to quantify, and additional evidence is required. Moreover, the experimental details were insufficient, including the number of animals, feeding duration, and modeling indicators, undermining the reliability of the conclusions. Interestingly, PDHA1 may also have therapeutic potential, but its mechanism and pathways remain unclear (156).

Another hypothesis links Cu imbalance to iron (Fe) deficiency (157). Epidemiological studies have shown that DM incidence is higher in individuals with low iron levels (158). This may result from decreased ceruloplasmin during Cu deficiency. As the primary serum Cu carrier, ceruloplasmin oxidizes Fe^2+^ to Fe^3+^ and, together with transferrin, participates in iron transport. Cu deficiency may thus disrupt iron metabolism (159), promoting DM progression. However, the mainstream view associates DM with iron excess, which induces oxidative stress and ferroptosis (160). Although ceruloplasmin is involved in iron metabolism, this hypothesis remains speculative, lacking molecular and mechanistic evidence.

Additionally, a multiple linear regression analysis of over 2000 subjects revealed a positive correlation between white blood cell count and Cu levels (p = 0.013). Sensitivity analysis validated these findings, suggesting that excessive serum Cu may elevate blood glucose via inflammatory markers and introducing a new mediator linking Cu to DM pathogenesis (161). However, current research is limited to clinical observation and data analysis without direct causal evidence.

Zinc (Zn) metabolism also plays an important role in DM mechanisms. DM increases urinary Zn excretion, reducing serum Zn levels, while epidemiological studies show that Zn supplementation can slow DM progression. Excess Cu may displace Zn from enzymes, exacerbating oxidative stress (162). These findings emphasize that Cu dysregulation mediates T2DM initiation and progression through β-cell damage, metabolic reprogramming, and trace element interactions.

The relationship between insulin resistance and Cu metabolism has also been investigated. A study of over 300 women found that Cu levels were lower in the insulin-sensitive group compared to the insulin-resistant group, and serum Cu was positively correlated with HOMA-IR (94, 163). Although limited to women, and hormonal effects cannot be excluded, these findings remain informative. Higher Cu levels in metabolic syndrome (MS) patients are also associated with insulin resistance (164), further supporting a positive correlation between serum Cu and insulin levels within certain ranges, possibly linked to obesity and lipid metabolism abnormalities. Cu can also activate insulin signaling independently of insulin and insulin receptor regulatory factors (InR) (165). The phosphatase and tensin homolog (PTEN) protein is a negative regulator of insulin signaling, and Cu reduces PTEN levels in mouse adipocytes, enhancing insulin resistance (166). Additionally, Cu inhibits IDE activity, preventing extracellular insulin degradation and increasing insulin levels (167).

Current research on insulin and Cu metabolism is limited, and the positive correlation remains inconclusive. Findings indicate that high Cu elevates insulin levels while reducing its activity, whereas physiological insulin clears excess Cu to reduce its toxicity. This does not contradict the harmful effects of Cu toxicity or the protective role of physiological insulin.

#### 3.1.2 T1D

Unlike T2D, the primary pathological mechanism of T1D is the absolute insulin deficiency caused by complete pancreatic β-cell dysfunction. Notably, the relationship between T1D and Cu dysregulation differs from that of T2D (99). A review suggested that changes in Cu levels did not occur in the early stages of T1D (100), as no significant differences existed in blood Cu levels between children with T1D and healthy controls (101). However, this study focused on Cu content in red blood cells, which does not accurately reflect circulating Cu levels. Animal studies have shown that insulin deficiency or defects in the insulin/protein kinase B (Akt2) pathway decrease ATP7A-SOD3 activity, resulting in intracellular Cu accumulation (92, 168). Given that insulin deficiency occurs in the early stages of T1D, Cu dysregulation induced by insulin deficiency should also appear during this initial phase. Physiological insulin clears excess Cu, which does not conflict with elevated insulin levels due to high Cu exposure. This distinction clarifies differences between physiological mechanisms and pathological effects. Furthermore, studies indicate that sustained hyperglycemia stimulates mitochondria to produce excessive ROS, triggering non-enzymatic glycation of proteins, lipids, and nucleic acids (102). Accumulation of advanced glycation end-products causes Cu release from protein-bound sites (85), raising blood Cu and plasma ceruloplasmin levels, and generating additional ROS, which damages pancreatic β-cells (92, 103). A recent animal study found that rats exposed to Cu at twice the recommended daily dose exhibited significantly higher blood glucose and weight loss compared with pure T1D rats, confirming that high Cu can exacerbate hyperglycemia and DM symptoms (169). Experimental results also showed elevated ROS, H_2_O_2_, and inflammatory cytokines (IL-6) in perivascular adipose tissue (PVAT), suggesting oxidative stress and inflammatory mechanisms underlying high Cu–induced vascular injury in DM (161). However, this study did not compare critical DM markers such as blood insulin or C-peptide levels. Additionally, the control group received saline injections only, without a control group exposed to normal Cu levels for comparison.

Since T1D primarily occurs in children and adolescents, observational research found that children with T1D had significantly higher hypercupremia compared to healthy children, and HbA1c correlated positively with plasma Cu levels (170). Sakhr et al. observed significantly elevated Cu and Cu/Zn ratios in children with T1D complicated by attention-deficit/hyperactivity disorder (ADHD) compared to children with ADHD alone (P<0.05), with HbA1c positively associated with the Cu/Zn ratio (105). Interestingly, two gender-specific studies observed elevated circulating Cu levels positively correlated with HbA1c in adult males with T1D, but not in females. Both studies found a negative correlation between HbA1c and Zn/Cu ratios in patients (96, 171). Lack of elevated circulating Cu in females might be related to estrogen, progesterone, or Zn metabolism abnormalities.

Collectively, these studies strongly suggest a bidirectional relationship between Cu dysregulation and hyperglycemia, with elevated glucose potentially causing Cu imbalance through glycation. Such interactions likely exist in all DM types and associated complications. Timely and effective glycemic control remains a reliable approach to mitigate Cu dysregulation.

#### 3.1.3 Gestational diabetes mellitus

Researchers have extensively explored the close correlation between circulating Cu levels and gestational diabetes mellitus (GDM). Zheng et al. reported that each quartile increase in plasma Cu correlated with an average glucose increase of 2.7 mg/dL in pregnant women (107). Higher plasma Cu levels in GDM compared to normal pregnancies appear more pronounced in Asian populations (108), possibly due to lower dietary intake of Cu-rich foods (e.g., red meat, milk) in Asians compared to Caucasians, making geographic research particularly relevant. Multiple generalized linear model analyses involving over 20,000 pregnant women collectively indicate that elevated Cu concentrations in early pregnancy increase GDM risk (172, 173). However, in one study, women with GDM had significantly higher age, parity, gravidity, and pre-pregnancy body mass index (BMI) compared to controls (P<0.001). While these factors indeed increase GDM risk, their presence introduces confounding limitations.

Another GLM analysis involving over 4,800 pregnant women found that white blood cell count, neutrophil count, and platelet count positively correlated with circulating Cu levels and pregnancy-related DM risk (P<0.05), suggesting inflammatory markers mediate the Cu-GDM association (174). Unfortunately, no animal or cellular experiments have investigated specific inflammatory mechanisms. Women with T1D complicated by preeclampsia (PE) exhibited significantly elevated Cu/Zn and Cu/high-density lipoprotein (HDL) ratios (109), suggesting these indices may serve as predictive markers for T1D-PE.

Conversely, low Cu levels are not always beneficial; Cu deficiency in GDM may also lead to severe consequences. A retrospective cohort study of 8,169 pregnant women revealed that each 50% increase in Cu concentration within safe limits during early pregnancy reduced GDM risk by 25% in underweight women (111). This highlights the importance of monitoring Cu deficiency risks in malnourished women with early GDM, suggesting appropriate Cu supplementation may be beneficial.

In summary, excessive circulating Cu frequently accompanies increased BMI, while low circulating Cu often accompanies malnutrition, both representing significant risk factors for GDM. Differences among study findings may reflect variations in nutritional status of GDM patients. Improved measurement techniques and rigorous control of confounding factors may help resolve these discrepancies.

### 3.2 Cu dysregulation and complications of DM

#### 3.2.1 Diabetic cardiomyopathy

DCM is a severe complication caused by hyperglycemia (175). Low expression of the CTR1 gene in myocardial cells leads to insufficient intracellular Cu uptake and subsequent Cu deficiency in DCM patients (176). This reduces the activity of Cu-sensitive SOD3, impairing its ability to neutralize superoxide radicals. Consequently, oxidative stress-induced myocardial fibrosis, functional loss, and apoptosis occur (112, 113). Additionally, reduced intracellular Cu causes a deficiency or functional impairment of lysyl oxidase (LOX) and significantly reduces CCO activity (114). The inhibition of LOX-mediated cross-linking of type I and III collagen fibers leads to decreased toughness and elasticity of connective tissue. This results in impaired left ventricular systolic function and compensatory myocardial contraction, ultimately contributing to concentric myocardial hypertrophy (115). As a key component of mitochondrial respiratory chain complex IV, CCO serves as the catalytic core of the oxidase complex and plays an essential role in oxidative phosphorylation. Reduced CCO activity impairs electron transfer in the mitochondrial respiratory chain, suppresses mitochondrial function and significantly lowers ATP levels. This ultimately causes myocardial hypoxia and impairs cardiac energy metabolism (177). Simultaneously, extracellular Cu accumulation resulting from impaired Cu uptake acts as a catalyst for DCM progression (116). Cu toxicity disrupts redox balance, activates inflammatory reactions, and promotes excessive ROS and advanced glycation end products (AGEs), leading to abnormal myocardial structure and function (117, 118), ultimately exacerbating DM-induced cardiomyopathy. Myocardial ischemia associated with this process may further lead to myocardial infarction (119). The studies mentioned above collectively indicate that uneven Cu distribution resulting from CTR1 dysfunction, characterized by intracellular Cu deficiency and extracellular Cu excess, is essential in DCM pathogenesis. The mechanism of Cu dysregulation in DCM-induced myocardial hypertrophy is different from myocardial hypertrophy caused by systemic Cu deficiency alone.

Sustained hyperglycemia-induced cuproptosis and myocardial apoptosis are also critical factors in DCM development (178). High glucose levels stimulate circulation of AGEs, activating transcription factor 3–transcription factor PU.1 and increasing Cu transporter (solute carrier family 31 member 1) expression (179). This enhances myocardial Cu accumulation, causing Fe-S cluster protein depletion and mitochondrial ROS accumulation, resulting in myocardial cuproptosis (36). Animal experiments found that hyperglycemia reduces myocardial Cox11 and mitochondrial Cox17 content, potentially blocking mitochondrial Cu binding (mtCoI) (180). Restoring Cu transport proteins such as Cox17 and mitochondrial Cu enzymes improved cardiac function in DM-induced heart failure in rats. However, experimental details such as total rat numbers, grouping, modeling success criteria, and treatment durations were inadequately described, compromising the reliability of conclusions.

Moreover, excessive myocardial intracellular Cu can upregulate LOX, accelerate collagen fiber cross-linking, induce myocardial fibrosis, and exacerbate myocardial injury (181). These findings indicate severe myocardial damage from excessive intracellular Cu, consistent with previous studies linking intracellular Cu reduction with DCM. Thus, precise control of myocardial Cu concentrations within a suitable range is critical, as deviations in either direction cause pathological outcomes.

#### 3.2.2 Diabetic kidney disease

Diabetic kidney disease (DKD) is a prevalent renal complication of DM (122), in which oxidative stress plays a central pathogenic role (123). Cu deposition in renal tissues induces oxidative stress through downregulation of anti-apoptotic genes [B-cell lymphoma-2 genes (BCL2)], upregulation of pro-apoptotic genes (BCL2-antagonist/killer 1, BCL-2-associated X protein), and increased expression of apoptosis proteins (Caspase-9, Caspase-3) (124). These changes cause mitochondrial injury, granular degeneration, and vacuolar degeneration of renal tubular epithelial cells (125), leading to proximal tubular necrosis, renal fibrosis (126), persistent albuminuria, reduced glomerular filtration rate, and elevated blood pressure (127).

In DM rats, elevated renal MT and plasma CP levels indicate increased renal Cu accumulation (128). Mutations in the ATP7A and ATP7B genes impair Cu excretion, further increasing renal Cu. Although urinary Cu is typically low, early glomerular basement membrane damage may increase urinary CP levels (129). A cross-sectional study indicated that elevated urinary CP is an independent predictor of poor DKD prognosis, closely associated with renal function deterioration (130). This suggests increased urinary CP reflects renal Cu excretion due to compensatory failure, indicating renal Cu overload and tissue injury.

A recent study found that cardiopulmonary bypass significantly elevated blood and urinary Cu, increased circulating cuproptosis-specific proteins (heat shock protein 70, DLAT), and reduced expression of renal cuproptosis-related proteins (lipoic acid synthase, aconitase 2, succinate dehydrogenase complex iron-sulfur subunit B) in diabetic rats (182). Renal mitochondrial structural damage was also observed. These results suggest cuproptosis contributes to acute kidney injury following cardiopulmonary bypass in DM, although the specific pathways and mechanisms remain unclear. Despite a well-designed and clearly described experimental process, the small sample size (104 clinical patients and 15 rats divided into three groups) limits the robustness of conclusions. Additionally, accidental losses during DM rat feeding or cardiopulmonary bypass procedures might further reduce effective sample sizes, weakening experimental validity.

#### 3.2.3 Diabetic retinopathy

DR is the most common microvascular complication of DM (183). Unlike other DM complications primarily caused by Cu overload, DR pathogenesis involves two types of Cu dysregulation (132). Retinal Cu deficiency reduces SOD1 activity, impairs antioxidant defense, and decreases activity of selenium-dependent glutathione peroxidase (GP) (133). Animal experiments reported reduced retinal Cu/Zn SOD activity in DR rats, causing mitochondrial oxidative injury, morphological swelling, and blurred mitochondrial cristae boundaries (184). This led to thinning of retinal ganglion cell layers, reduced cell number, blurred nuclear membranes, and nuclear disintegration, highlighting mitochondrial damage as a key intermediate mechanism in DR due to Cu protein dysfunction. However, these studies used only six rats per group, indicating insufficient sample sizes and low repeatability. Other studies found that berberine increased retinal ATP7A expression and intracellular Cu content, enhancing antioxidant systems (GSH, MT) to alleviate oxidative stress and improve DR lesions (134, 185). Additionally, hyperglycemia inhibits mitochondrial electron transport chain complexes in retinal Müller cells (rMC-1) and bovine retinal endothelial cells (BREC), increasing superoxide production. Exogenous Cu/Zn SOD supplementation prevented these effects and reduced cell death (133).

Secondly, excessive serum Cu can damage retinal pigment epithelium, plexiform layers, and optic nerve fibers (135). Du et al. demonstrated that excess Cu worsened diabetic retinopathy by elevating oxidative stress under hyperglycemic conditions (133). Recent case-control studies reported significantly elevated serum Cu levels in DR patients compared to DM patients without DR (186). Another study found Cu overload downregulated retinal microtubule genes and caused developmental defects in zebrafish embryos (136).

Collectively, these findings indicate that elevated circulating Cu, intracellular Cu deficiency, and hyperglycemia collectively contribute to retinal damage and DR progression, paralleling Cu dysregulation mechanisms in DCM. Investigating Cu-related genes may offer novel insights into DR pathogenesis.

#### 3.2.4 Diabetic peripheral vascular disease

DPVD is a common and severe vascular complication in DM patients (187). Cu accumulation in vascular smooth muscle cells (VSMCs) accelerates collagen fiber cross-linking, promoting fibrosis by activating LOX expression and catalytically degrading endothelium-derived relaxing factors (nitric oxide and its derivatives) (114). Meanwhile, insulin deficiency or defects in the insulin/Akt2 pathway mediate downregulation of endothelial ATP7A, impairing the binding of Cu to SOD3 in the trans-Golgi network (91). This increases vascular O^2-^ production, causing endothelial dysfunction (138), basement membrane thickening, and microthrombosis, ultimately resulting in chronic vasoconstriction accompanied by elevated pro-inflammatory cytokines (169). Additionally, extensive glycosylation of elastin and collagen in arterial walls increases their affinity for Cu, exacerbating intracellular Cu accumulation and worsening DPVD (58).

A recent experimental study demonstrated marked cuproptosis (increased serum Cu concentrations and reduced Fe-S cluster proteins) in diabetic mice with limb ischemia and high-glucose/nutrient-deprived human microvascular endothelial cells (188). Treatment with the Cu chelator ammonium tetrathiomolybdate effectively alleviated hindlimb ischemic injury and reduced cuproptosis-related cell death, suggesting cuproptosis as a therapeutic target for diabetic limb ischemia. However, the femoral artery ligation used in the mouse model does not accurately reflect the causal relationship between DM and peripheral vascular disease. Additionally, the significance of nutrient deprivation in cells was unclear, and direct evidence linking high glucose to cell cuproptosis was lacking.

The findings, when considered as a whole, indicate that an excess of copper in vascular endothelial cells promotes DPVD by means of mediating copper protein imbalance and copper death, a process that is facilitated by hyperglycemia and insulin deficiency.

## 4 Clinical intervention

Given that Cu dysregulation in DM primarily manifests as excessive circulating Cu and insufficient intracellular Cu, it not only induces metabolic disorders but also triggers cell death processes (cuproptosis, oxidative stress, inflammation), leading to organ damage. Current therapeutic strategies mainly focus on reducing Cu bioavailability (chelators, Zn) or mitigating its downstream effects (nanoparticles). Since insulin and various hypoglycemic drugs affect Cu metabolism through multiple mechanisms, they are discussed separately.

### 4.1 Hypoglycemic drugs and insulin

Insulin and hypoglycemic medications remain mainstream DM treatments. Therefore, the relationship between their mechanisms of hypoglycemia, organ protection, and regulation of Cu metabolism is widely studied. Experimental evidence indicates that exogenous insulin supplementation under conditions of insulin deficiency can regulate glycosylation of proteins involved in the ATP7A-SOD3 axis and Cu homeostasis (92, 168). Insulin activates Akt2, inhibits protein kinase A (PKA), elevates ATP7A expression, restores SOD3 activity, reduces intracellular Cu, and re-establishes physiological Cu balance and energy metabolism. This highlights insulin’s positive regulatory effects on Cu metabolism. Additionally, pioglitazone, a widely used hypoglycemic drug, reportedly increases SOD3 levels (189). Cell and animal studies demonstrated that pioglitazone interacts with Cu ions, activates AMP-activated protein kinase (AMPK) phosphorylation, induces autophagy, reduces mitochondrial ROS, and suppresses inflammatory cytokines (IL-1β and tumor necrosis factor-α (TNF-α)) (190–193). The inclusion of rescue experiments enhances the reliability of these findings. Pioglitazone likely positively regulates Cu balance by restoring impaired SOD3 function in DM, thus addressing Cu dysregulation, oxidative stress, and inflammation. Metformin, a first-line T2D drug, exerts hypoglycemic effects partly through mitochondrial Cu chelation (194). *In vitro* and epidemiological studies revealed that metformin forms stable complexes with Cu, significantly reducing circulating Cu levels in users (195, 196). Recent animal studies showed that metformin restores SOD activity in T2D mice by decreasing serum GSH, elevating oxidized glutathione (GSSG), and enhancing antioxidant defenses (197). It also decreases pancreatic tissue expression of IL-6 and TNF-α, increases anti-inflammatory IL-10, and alleviates DM-associated chronic inflammation. Metformin’s restoration of SOD suggests an additional Cu-regulatory mechanism beyond simple Cu chelation.

The cardioprotective effects of dapagliflozin are well-established (198). Recent studies indicate that dapagliflozin alleviates myocardial fibrosis post-myocardial infarction by inhibiting the HIF-1α/transforming growth factor-β (TGF-β) pathway, reducing Cu toxicity markers, clearing ROS, and lowering Cu concentrations (199).

These findings suggest that, although hyperglycemia and Cu dysregulation mutually exacerbate each other, correction of Cu imbalance by hypoglycemic drugs is not solely attributed to glucose-lowering effects but reflects distinct pharmacological properties. Current mechanisms mainly involve restoring pathologically reduced SOD3 levels and directly chelating excess Cu. Although not the primary mode of action for these drugs, maintaining Cu homeostasis provides new therapeutic insights. Cu balance, as an independent mechanism, significantly contributes to reducing blood glucose and alleviating DM-related tissue damage.

### 4.2 Chelation therapy

Cu dysregulation in DM and its complications is predominantly characterized by Cu excess, which causes tissue and organ damage through Cu toxicity and cuproptosis. Therefore, prevention and reversal using Cu chelating agents is emerging as a novel therapeutic strategy (116, 200). Copper chelation and copper-ionophores are being tested in cancer, and the translational lessons might apply to diabetes as well (201, 202). For instance, tetrathiomolybdate (TTM) improves insulin resistance and restores glucose tolerance in T2D model mice by reducing serum Cu and ROS levels (203). Penicillamine chelates Cu, preventing high glucose- and Cu-induced downregulation of mitochondrial fusion protein 2 (MFN2), endoplasmic reticulum (ER) stress, and inflammation in retinal pigment epithelial cells, thereby protecting mitochondria and reducing inflammation (135). Recent research identified a geometric isomer of a new compound, 1-methylimidazole-2-formol nicotinic hydrazide (X1NIC), which demonstrates strong Cu chelation and may serve as a chemical tool to regulate abnormal metal-peptide interactions in T2D pathogenesis (204). Under adequate dosage, the AGE inhibitor aminoguanidine, as a weak chelating agent, reduces Cu concentration by promoting urinary and biliary excretion of free or weakly bound Cu, thereby decreasing AGE formation and proteinuria in DM rats. Angiotensin receptor blockers and aldose reductase inhibitors also inhibit AGE formation in DM via Cu chelation (205). Continuous low-dose chelation therapy shows considerable potential as a clinical approach to prevent and manage DM complications. Triethylenetetramine (TETA) has been extensively studied in DCM; experiments demonstrated that it reverses peroxidase upregulation, increases ATP7A and CCS expression, restores left ventricular Cu uptake, enhances Cox17–mtCoII colocalization, normalizes Cox11 expression, restores mitochondrial integrity, reduces serum Cu, increases urinary Cu excretion, and improves left ventricular structure and function in streptozotocin (STZ) rats (119, 180). Zn, as a competitive ion for Cu absorption and circulation, also participates in DM therapy by chelating Cu to reduce circulating Cu levels (142).

Although these experimental results support the potential of traditional Cu chelating agents to mitigate DM and its complications, their suitability for clinical application in DM patients requires further evaluation. Currently, no clinical data are available. Future studies must focus on side effects, safety, and availability, or on developing Cu chelation as a primary mechanism for novel therapies targeting DM and its complications.

### 4.3 Nanoparticles

In recent years, the integration of nanotechnology and Cu-based molecules has rapidly advanced the treatment of DPVD. Although not directly related to excessive circulating Cu, the combination of Cu’s broad-spectrum antibacterial properties with the targeted delivery of nanoparticles offers a novel therapeutic option. Nanocomposite membranes containing n-CuO_2_ or CuO nanoparticles (NPs) release Cu^2+^ at wound sites, mediating antibacterial and anti-inflammatory effects, combating multidrug-resistant pathogens, enhancing angiogenesis, and promoting tissue regeneration and healing in DM-related injuries. These approaches hold promise for repairing chronic wounds in DM, where extracellular matrix function is impaired, angiogenesis is reduced, and susceptibility to inflammation and infection is increased (206).Low-concentration safety and efficacy have been confirmed in promoting granulation tissue development in DM animal models and normal human skin fibroblast cell lines (207), but these therapies remain at the preclinical stage, and their long-term toxicity requires careful evaluation.

## 5 Future directions and outstanding questions

1. Current clinical and animal studies have preliminarily established a correlation and interaction between Cu dysregulation, DM, and its complications. However, more precise regulatory factors and definitive pathways require exploration. Human studies are needed to verify findings from rodent models. Exploring deeper mechanisms is the immediate research priority.
2. Approximately 25% of T2D patients have comorbid depression (208). It has been proposed that Alzheimer’s disease (AD) with insulin resistance and T2D represents “type 3 DM” (209). Cu, as the third most abundant transition metal in the brain, plays a critical role in cognitive function by clearing ROS and protecting neurons (210). Chronic Cu exposure or Cu dysregulation may be linked to neurodegenerative diseases (211). Research on Cu mechanisms in nervous system disorders is active, but discoveries linking Cu dysregulation and DM-related encephalopathy remain largely epidemiological, lacking molecular or mechanistic studies. Effective interventions are still under development. Targeted research in this field is anticipated.
3. Lysosomal autophagy acts as a cellular defense mechanism by clearing abnormal proteins and damaged organelles, preventing Cu-driven lipid accumulation, maintaining Cu homeostasis, and preventing Cu-induced apoptosis (29). Unlike general cellular autophagy, lysosomal autophagy ensures cell survival and plays a pivotal role in treating diseases caused by Cu overload. Although the interplay between Cu metabolism and lysosomal autophagy remains underexplored, drugs designed to activate this pathway could reduce Cu toxicity. Its high specificity and favorable tissue safety profile further support its potential clinical value. Therefore, targeting lysosomal autophagy emerges as one of the most promising therapeutic targets in copper-related diabetes interventions.
4. Specific Cu ion carriers may provide tissue-targeted therapy. For example, precisely delivering Cu carriers to myocardial or ocular tissues in DM patients with Cu deficiency could offer new treatment strategies for various DM complications.
5. Metformin reduces cancer incidence in DM patients (212, 213), while cuproptosis plays a key role in cancer pathogenesis (52–54, 214–216). Future research should clarify whether metformin’s anti-cancer effects are mediated by Cu chelation and whether Cu protein activity pathways contribute to its mechanism.
6. The clinical applicability of copper chelators in diabetic patients is yet to be fully evaluated, and the potential for adverse effects and safety concerns must be emphasised. The utilisation of nanoparticle therapy for the treatment of DPVD remains in the preclinical research stage. Further in-depth studies are required to ascertain the long-term toxicity of the therapy.
7. Non-enzymatic glucose sensors based on Cu and its oxides show potential as novel biomarkers due to their stability, ease of preparation, and high sensitivity (217). They may become powerful tools for next-generation glucose assays.

## 6 Conclusions

This article systematically reviewed the role of Cu dysregulation, including Cu excess, Cu deficiency, and uneven Cu distribution, in DM and its complications. Cu toxicity arises mainly through dysregulated expression of Cu-related proteins, mediating oxidative stress, cuproptosis, and insulin/glucose dysregulation to damage tissues and organs. Hyperglycemia further amplifies this damage. Notably, cuproptosis is a newly proposed mechanism, and most evidence remains preliminary. The experimental findings and their significance should therefore be interpreted cautiously to avoid overstatement. With advances in lipoprotein-cuproptosis research in oncology, precision medicine approaches such as nanoparticles and targeted therapies, and emerging research on Cu metabolism, new perspectives and strategies will likely arise for understanding the molecular mechanisms and treatment of DM.
